# Genome-wide evidence for positive selection and recombination in *Actinobacillus pleuropneumoniae*

**DOI:** 10.1186/1471-2148-11-203

**Published:** 2011-07-13

**Authors:** Zhuofei Xu, Huanchun Chen, Rui Zhou

**Affiliations:** 1 Division of Animal Infectious Disease, State Key Laboratory of Agricultural Microbiology, College of Veterinary Medicine, Huazhong Agricultural University, Wuhan 430070, China

## Abstract

**Background:**

*Actinobacillus pleuropneumoniae *is an economically important animal pathogen that causes contagious pleuropneumonia in pigs. Currently, the molecular evolutionary trajectories for this pathogenic bacterium remain to require a better elucidation under the help of comparative genomics data. For this reason, we employed a comparative phylogenomic approach to obtain a comprehensive understanding of roles of natural selective pressure and homologous recombination during adaptation of this pathogen to its swine host.

**Results:**

In this study, 12 *A. pleuropneumoniae *genomes were used to carry out a phylogenomic analyses. We identified 1,587 orthologous core genes as an initial data set for the estimation of genetic recombination and positive selection. Based on the analyses of four recombination tests, 23% of the core genome of *A. pleuropneumoniae *showed strong signals for intragenic homologous recombination. Furthermore, the selection analyses indicated that 57 genes were undergoing significant positive selection. Extensive function properties underlying these positively selected genes demonstrated that genes coding for products relevant to bacterial surface structures and pathogenesis are prone to natural selective pressure, presumably due to their potential roles in the avoidance of the porcine immune system.

**Conclusions:**

Overall, substantial genetic evidence was shown to indicate that recombination and positive selection indeed play a crucial role in the adaptive evolution of *A. pleuropneumoniae*. The genome-wide profile of positively selected genes and/or amino acid residues will provide valuable targets for further research into the mechanisms of immune evasion and host-pathogen interactions for this serious swine pathogen.

## Background

In the evolutionary history of many microorganisms, positive selection and homologous recombination are two indispensable driving forces for adaptation to new niches. Both of them contribute to the genetic variations that might influence the population diversification and adaptation of pathogenic microorganisms [1,2]. Recent studies on the genome-wide evolutionary dynamics have highlighted the important roles of selection and recombination in the molecular evolution of bacterial pathogens, including *Escherichia coli *[1], *Listeria monocytogenes *[3], *Salmonella *spp. [4], *Streptococcus *spp. [5], and *Campylobacter *spp. [6]. These analyses have revealed that a certain number of protein-coding genes subject to natural selection pressure are usually involved in the dynamical interactions between host and pathogen, especially in the immune and defense-associated functions [1]. Diversifying selection operating on these genes may be caused by pathogen-host co-evolutionary arms race [7,8].

In the present study, *d*_N_/*d*_S_-based methods were applied to detect evidence of genome-wide positive Darwinian selection. Estimating the ratio (*ω*) of the rate of nonsynonymous nucleotide substitutions (*d*_N_) to that of synonymous substitutions (*d*_S_) is a powerful approach for measuring selective pressure on the protein-coding level: *ω *= 1, < 1, > 1 indicate neutral evolution, purifying (negative) selection, and positive (adaptive) selection, respectively [9,10]. The codon models further developed by Nielsen and Yang allow variation in *ω *among sites [11], which have an extensive capability to find evidence for adaptive evolution in most functional genes where only a small fraction of amino acid sites are subject to strong positive selective pressure [12]. Thus far this approach has been widely used for genome-wide selection analyses in pathogenic viruses, bacteria, and eukaryotes [9,13]. A substantial number of genes encoding highly variable antigens are identified to undergo adaptive selection particularly on some functional sites for evasion of host immunity [1,4,14].

*Actinobacillus pleuropneumoniae*, a Gram-negative coccobacillus belonging to the *Actinobacillus *genus of *Pasteurellaceae *family, is a strictly swine pathogen and colonizes in the upper respiratory tract of porcine [15]. This pathogen has caused an economically severe disease characterized by pulmonary lesions, pleuritis, and pericarditis in pigs [16]. According to the differences in capsular polysaccharides, *A. pleuropneumoniae *has been divided into 15 serovars [17]. The recent comparative genomics studies through both high-throughput approaches of genome sequencing and microarray have depicted the compositions of the pan-genome and confirmed the contribution of genes loss or gain to the diversity in virulence and serovar of *A. pleuropneumoniae *[18,19]. However, besides large genetic variations resulting from DNA acquisition and genome reduction, small sequence differences occurring in the conserved genes, including point mutations, insertions/deletions (indels), and intragenic recombination, may also play a crucial part in the alteration of antibiotic resistance, pathogenicity and immunogenicity [20,21]. But to date, no research pays enough attention to the linkages between genetic alterations and putative functional roles in intraspecies conserved genes of *A. pleuropneumoniae *at the whole genome level.

In order to further trace evolutionary trajectories on the core genome of pathogenic bacterium *A. pleuropneumoniae*, we employed a genome-wide analyses approach to investigate the effects of natural selection and homologous recombination operating on the coding genes. Our analyses focused on the evolutionary characterizations of core genome genes that are shared by 12 *A. pleuropneumoniae *genomes. Many genes were shown to be under strong positive selective pressure and primarily associated with the fitness and immunogenic properties of this swine pathogen.

## Methods

### Genome dataset and alignment

Twelve genome sequences of *A. pleuropneumoniae *were retrieved from NCBI Genome database (http://www.ncbi.nlm.nih.gov/genome). The sequences included 3 complete genomes and 9 draft genome assemblies (see details in Table 1). Orthologous gene content information and annotation with COG functional classification have been defined in our recent work and used here [19]. To increase accuracy and power of selection analyses, an ortholog set was excluded if it satisfied any of the following criteria: the length of any gene lower than 80% of the maximum length, more than one gene from each genome or less than four sequences. Protein-coding sequences longer than 50 codons were used in this study. Subsequently, the orthologous protein sequences were aligned using a progressive method implemented in T-Coffee v8.93 [22].

**Table 1:** Genome sequences of *A. pleuropneumoniae *used in this study

Strain	GenBank accession no.	Genome size (Mbp)	No. of CDS (> 49 codons)	Reference
JL03	CP000687	2.24	2,101 (2,035)	[60]
L20	CP000569	2.27	2,137 (2,072)	[61]
Ap76	CP001091	2.33	2,203 (2,134)	
4074	ADOD00000000	2.26	2,180 (2,101)	[19]
S1536	ADOE00000000	2.22	2,137 (2,061)	[19]
M62	ADOF00000000	2.27	2,223 (2,151)	[19]
Femφ	ADOG00000000	2.31	2,219 (2,138)	[19]
CVJ13261	ADOI00000000	2.26	2,204 (2,124)	[19]
D13039	ADOJ00000000	2.27	2,168 (2,091)	[19]
56153	ADOK00000000	2.27	2,195 (2,120)	[19]
1096	ADOL00000000	2.19	2,096 (2,033)	[19]
N273	ADOM00000000	2.25	2,149 (2,086)	[19]

Frameshift mutations (indels of a number of nucleotides not divisible by three) can lead to high nonsynonymous substitution rates, resulting in more false positive results when positive selection was estimated based on *d*_N_/*d*_S _ratios [5]. To avoid incorrect indels in the codon alignments, multiple sequence alignments were initially performed with amino acid sequences from each gene cluster, followed by conversion to the corresponding codon alignments using custom Perl scripts. The coding sequences located at the beginning or end of the contigs appeared to be more prone to frameshift sequencing errors. Therefore, we further assessed the quality for each alignment through obtaining the following information: overall identity, and identity in the first 30 nt and last 30 nt per alignment. The codon alignment sequences that contain frameshift mutations were checked and edited manually in the software MEGA4 [23] if identity is low.

### Calculation of *d*_N_, *d*_S_, codon bias, nucleotide diversity and informative sites

According to the method as defined by Nei and Gojobori [24], the number of synonymous nucleotide substitutions per synonymous site (*d*_S_) and the number of nonsynonymous nucleotide substitutions per nonsynonymous site (*d*_N_) were estimated for the resulting gene alignments using the program SNAP [25]. Gene-by-gene number of informative sites and genetic diversity were obtained from the output of the PhiPack program [26]. The analyses for the codon usage variation was performed by computing the effective number of codons (N_c_), which is a general measure of bias from equal codon usage in a gene. The N_c _value ranges from 20 for the strongest bias (where only one codon is used for each amino acid) to 61 for no bias [27,28]. The calculation of N_c _were implemented in the program CodonW 1.4 (http://codonw.sourceforge.net/).

### Detection of recombination events

Since recombining fragments among aligned codon sequences have a profound effect on the detection of the positively selective evidence [29], we first tested for recombination signals between sequences in the alignment of orthologous genes. Four statistical procedures GENECONV [30], pairwise homoplasy index (PHI) [26], maximum *χ*^2 ^[31] and neighbor similarity score (NSS) [32] were applied to discover the homologous recombination signals. Besides GENECONV version 1.81, the other three methods were implemented in the PhiPack package [26]. For the analyses of GENECONV, the parameter g-scale was set to 1, which allows mismatches within a recombining fragment. The *p*-values for inner fragments using 10,000 random permutations were used to indicate the significance of putative recombinant regions. For maximum *χ*^2^, a fixed window-size of 2/3 the number of polymorphic sites was used. For PHI, the window size was set to 100 nucleotides. Simulated *p*-values were estimated based on 1,000 permutations for PHI, maximum *χ*^2 ^and NSS.

### Detection of Selection

Maximum likelihood (ML) phylogenetic trees were reconstructed for each gene in the dataset of the core genome genes using the PhyML program [33]. A general time-reversible (GTR) model of nucleotide substitution with the ML estimates for gamma distributed rate heterogeneity of four categories (Г_4_) and a proportion of invariable sites were used in all tree reconstruction methods. The resulting topologies of ML trees were applied to the subsequent selection analyses.

To detect selective pressure acting on each coding gene, the rates of synonymous and nonsynonymous substitutions were estimated site-by-site using the *codeml *program from the PAML 4.2b package [34]. According to the topology of the resulting ML tree per gene alignment, two site-specific models that allow variable nonsynonymous (*d*_N_) and synonymous (*d*_S_) rate ratios (*ω *= *d*_N_/*d*_S_) among codons were applied to analyze our data set: M1a (NearlyNeutral) and M2a (PositiveSelection). Null hypothesis model M1a was nested with alternative selection model M2a. The latter model adds an extra site class for a fraction of positively selected amino acid sites with *ω *> 1; whereas models M1a only allows site classes with *ω *varying between 0 and 1 [10,35]. A likelihood ratio test (LRT) was carried out to infer the occurrence of sites subject to positive selective pressure through comparing M1a against M2a. Three replicates were run with *codeml *and the maximum likelihood values for each model were used in the LRT. The LRT statistic (twice the log-likelihood difference between the null and the alternative models) was compared with the *χ*^2 ^distribution with two degrees of freedom. The Bayes empirical Bayes approach was employed to identify positively selected sites under the likelihood framework [36].

### Mapping of positively selected sites to structure models of proteins

The web server PSORTb v3.0 was used to predict bacterial protein subcellular localization [37]. Integral beta-barrel outer membrane proteins were predicted by BOMP [38]. The three dimensional structure model of the protein encoded by the gene that showed evidence for positive Darwinian selection was modeled using the Phyre server [39]. The sites subject to positive selective pressure were mapped onto the structure and visualized by PyMol (http://www.pymol.org/).

### Statistical analyses

Multiple testing correction was performed to control for Type I errors according to the approach presented by Benjamini & Hochberg [40]. For all genes tested for recombination and positive selection, *q*-values were calculated from *p*-values using the R package *qvalue *with the proportion of true null hypothesis set to 1 (π_0 _= 1) [41]. A false discovery rate (FDR) of 10% was used for the recombination analyses. As the tests used for detecting positive selection was conservative [42], an FDR of 20% was set.

The non-parametric Mann-Whitney U-test was employed to determine the significance level for the differences among the selected continuous variables (i.e., *d*_N_, *d*_S_, codon bias and nucleotide diversity) between a given COG functional categories and all other categories. Binomial test was used to estimate association between each COG category and evolutionary forces (i.e. positive selection and/or homologous recombination); Bonferroni corrections for multiple comparisons were performed according to the number of one-sided tests. The significance level was set to 5% in this study. All statistic analyses were carried out using Perl scripts and R 2.11.1 [43].

## Results

### Properties of orthologous genes in 12 *A. pleuropneumoniae *genomes

In our recent work [19], 2,531 orthologous genes and 772 strain-specific genes have been identified in the pan-genome of 12 *A. pleuropneumoniae *strains using BlastClust. The above data set was used to further decode phylogenomic characterizations of pathogenic *A. pleuropneumoniae*. The evidence for homologous recombination and natural selection pressure whether operate on the conserved coding genes was estimated at the present study. After manually editing the aligned gene sequences and removing the low quality ones, a data set of sequence alignments of 1,960 orthologs was selected out, 81% (n = 1,587) of which were core genes that are present one copy per genome and the remaining (n = 373) were distributed genes present in at least four genomes.

The codon bias for each orthologous gene was measured by the effective number of codons (N_c _value) calculated by CodonW [28]. The reduction in N_c _indicates strong bias that significantly correlates with high gene expressivity [44]. *A. pleuropneumoniae *genes in the COG functional categories "Energy production and conversion", "Translation", "Amino acid transport and metabolism", "Nucleotide transport and metabolism", and "Carbohydrate transport and metabolism" were evident to have higher codon usage bias (*P *< 0.001, *P *< 0.001, *P *= 0.003, *P *= 0.001, and *P *< 0.001, respectively; one-tailed U-test) compared with genes in other COG categories. As is well known, genes bearing stronger codon bias are likely to be highly expressed and have housekeeping features [3,45]. So, high codon bias of genes present in the five COG categories is likely to elucidate the necessity of relevant coding products for implementing fundamental life cycle and essential physiological activities of *A. pleuropneumoniae*.

*A. pleuropneumoniae *genes in the functional categories "Replication, recombination and repair" and "Amino acid transport and metabolism" represented a tendency to have higher rates of synonymous (*d*_S_) nucleotide substitutions (*P *= 0.006 and *P *< 0.001, respectively; one-tailed U-test) in comparison with genes in other role categories (Table 2). On the other hand, genes in the functional categories "Replication, recombination and repair", "Amino acid transport and metabolism", "Coenzyme transport and metabolism" and "General functional prediction" showed a tendency to have higher rates of nonsynonymous (*d*_N_) substitutions (*P *= 0.012, *P *= 0.001, *P *= 0.007, and *P *= 0.007, respectively; one-tailed U-test) in comparison with genes in other COG categories (Table 2). Positive correlation was observed between *d*_S _and *d*_N _values for each COG category of *A. pleuropneumoniae *genes, indicating that natural selection might uniformly act on synonymous and nonsynonymous sites per gene. In addition, it was worth noting that the average *d*_S _and *d*_N _values were significantly lower (*P *= 0.001 and *P *< 0.001, respectively; one-tailed U-test) for genes in the COGs "Translation" than for genes in other COG categories. It has been suggested that genes involved in the translation machinery, e.g. ribosomal proteins and tRNA synthetases, usually evolved slowly with low *d*_S _and *d*_N_, probably due to structural and functional constraints required by the fundamental cell life cycle [20,46,47].

**Table 2:** The rates of synonymous (*d*_S_) and nonsynonymous (*d*_N_) nucleotide substitutions among different functional categories for *A. pleuropneumoniae *genes

COG category^a^	Number of genes analyzed	*d*_S _(± se)^b^	*d*_N _(± se)^c^	r ^d^
Energy production and conversion	114	44.2 × 10^-3 ^(± 5.2 × 10^-3^)	2.7 × 10^-3 ^(± 0.5 × 10^-3^)	0.78
Cell cycle control and cell division	26	59.7 × 10^-3 ^(± 13.0 × 10^-3^)	3.3 × 10^-3 ^(± 0.8 × 10^-3^)	0.69
Amino acid transport and metabolism	133	80.1 × 10^-3 ^(± 6.6 × 10^-3^)	4.1 × 10^-3 ^(± 0.4 × 10^-3^)	0.80
Nucleotide transport and metabolism	52	53.7 × 10^-3 ^(± 8.3 × 10^-3^)	2.5 × 10^-3 ^(± 0.4 × 10^-3^)	0.67
Carbohydrate transport and metabolism	109	56.8 × 10^-3 ^(± 6.5 × 10^-3^)	3.2 × 10^-3 ^(± 0.6 × 10^-3^)	0.73
Coenzyme transport and metabolism	92	59.5 × 10^-3 ^(± 7.7 × 10^-3^)	4.7 × 10^-3 ^(± 0.7 × 10^-3^)	0.57
Lipid transport and metabolism	32	61.4 × 10^-3 ^(± 11.3 × 10^-3^)	3.7 × 10^-3 ^(± 0.9 × 10^-3^)	0.75
Translation	151	42.9 × 10^-3 ^(± 4.5 × 10^-3^)	2.1 × 10^-3 ^(± 0.3 × 10^-3^)	0.58
Transcription	78	69.4 × 10^-3 ^(± 11.8 × 10^-3^)	3.8 × 10^-3 ^(± 0.8 × 10^-3^)	0.86
Replication, recombination and repair	94	83.3 × 10^-3 ^(± 11.8 × 10^-3^)	6.2 × 10^-3 ^(± 1.3 × 10^-3^)	0.69
Cell membrane and envelope biogenesis	120	63.3 × 10^-3 ^(± 10.8 × 10^-3^)	4.8 × 10^-3 ^(± 0.8 × 10^-3^)	0.85
Posttranslational modification, protein turnover, chaperones	84	55.3 × 10^-3 ^(± 7.2 × 10^-3^)	3.6 × 10^-3 ^(± 0.9 × 10^-3^)	0.75
Inorganic ion transport and metabolism	124	63.5 × 10^-3 ^(± 6.1 × 10^-3^)	3.9 × 10^-3 ^(± 0.4 × 10^-3^)	0.67
Secondary metabolites biosynthesis, transport and catabolism	12	34.3 × 10^-3 ^(± 11.4 × 10^-3^)	2.7 × 10^-3 ^(± 0.9 × 10^-3^)	0.90
General function prediction only	203	65.7 × 10^-3 ^(± 6.1 × 10^-3^)	4.6 × 10^-3 ^(± 0.5 × 10^-3^)	0.82
Function unknown	177	73.6 × 10^-3 ^(± 9.1 × 10^-3^)	4.7 × 10^-3 ^(± 0.6 × 10^-3^)	0.86
Signal transduction mechanisms	30	39.5 × 10^-3 ^(± 8.6 × 10^-3^)	2.0 × 10^-3 ^(± 0.4 × 10^-3^)	0.60
Intracellular trafficking, secretion and vesicular transport	47	66.4 × 10^-3 ^(± 16.5 × 10^-3^)	7.0 × 10^-3 ^(± 3.5 × 10^-3^)	0.85
Defense mechanisms	22	63.6 × 10^-3 ^(± 20.1 × 10^-3^)	3.9 × 10^-3 ^(± 1.5 × 10^-3^)	0.76
Not in COGs	258	56.1 × 10^-3 ^(± 5.9 × 10^-3^)	6.6 × 10^-3 ^(± 0.8 × 10^-3^)	0.72

^a ^Two COG functional categories including one gene (i.e. "RNA processing and modification" and "Extracellular structures") are not showed.

^b ^The average rate (*d*_S_) of synonymous substitutions (nucleotide mutations that do not alter the amino acid sequence) for all orthologous genes within a give COG role category is displayed, followed by the standard error that is represented in parentheses.

^c ^The average rate (*d*_N_) of nonsynonymous substitutions (nucleotide mutations that alter the amino acid sequence) for all orthologous genes within a give COG role category is displayed, followed by the standard error that is represented in parentheses.

^d ^Correlation coefficient between the values of *d*_S _and *d*_N_.

### A substantial number of genes showing evidence for recombination in the core genome of *A. pleuropneumoniae*

Among the 1,587 orthologous core genes, 2% (29 genes) had no occurrence of nucleotide substitutions and thus were not further investigated for evidence of homologous recombination. Furthermore, among the remaining genes, 197 gene alignments that contain few informative sites less than two could not be analyzed with programs in PhiPack and were removed from the ortholog sets. Finally, 86% of total core genes were selected to conduct the subsequent recombination analyses through four approaches. The evolutionarily conserved core genes (n = 226) were summarized (Additional file 1) and the biological functions carried out by their coding products may be essential for the survival of *A. pleuropneumoniae*. Notably, conserved genes were significantly enriched in the COG category "Translation" with a low Bonferroni corrected *p*-value (*P *< 0.001; Binomial test); this result was consistent with low *d*_S _and *d*_N _values mentioned before. These translation-associated protein-coding genes are generally involved in the fundamental cellular activity and thus hardly have any changes at the amino acid level as a result of functional constraints. Overall, among 12 *A. pleuropneumoniae *genomes, 822 orthologous core genes (52% of all 1,587 core genome genes) were identified to have significant evidence for recombination (FDR < 10%) that was detected by at least one of the four tests (Additional file 2). A total of 493, 675, 659 and 559 orthologs were identified to have recombination signals using GENECONV, Maximum *χ*^2^, NSS and PHI, respectively. Additionally, a total of 149, 148, 160, and 365 orthologs exhibiting recombination signals were identified by using one, two, three, and all four recombination tests, respectively.

**Figure 1:**
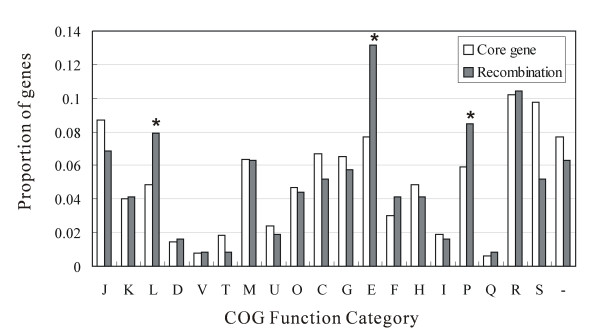
**Genes with evidence of recombination are enriched in three COG functional categories**. The abscissa represents different COG functional categories. The ordinate represents the proportion of genes in each COG category. Bars in dark gray stand for proportions of genes (n = 365) with evidence for recombination (FDR < 10%). Bars in white stand for proportions of all core genes (n = 1,587) of *A. pleuropneumoniae *used in this study. Asterisks mark certain COG categories that significantly enriched with recombining genes (*P *< 0.05, Binomial test). The COG categories are coded as follows: J, translation; K, transcription; L, DNA replication, recombination and repair; D, cell division and chromosome partitioning; V, defense mechanisms; T, signal transduction; M, cell wall/membrane biogenesis; U, intracellular trafficking, secretion and vesicular transport; O, posttranslational modification, protein turnover and chaperones; C, energy production and conversion; G, carbohydrate transport and metabolism; E, amino acid transport and metabolism; F, nucleotide transport and metabolism; H, coenzyme metabolism; I, lipid metabolism; P, inorganic ion transport and metabolism; Q, secondary metabolites biosynthesis, transport and catabolism; R, general functional prediction only; S, function-unassigned conserved proteins; -, unknown proteins not in the COG collection.

It is worth noting that 23% of all core genes, which were selected as recombinants by all four methods for testing recombination, have more informative sites (*P *< 0.001; one-sided U-test) and higher nucleotide diversity (*P *< 0.001; one-sided U-test). For all core genome genes, association between COG categories and the number of genes with recombining fragments was estimated (Figure 1). Core genes that exhibit evidence for recombination were significantly overrepresented in three COG categories "Replication, recombination and repair", "Amino acid transport and metabolism", and "Inorganic ion transport and metabolism" (uncorrected *P *= 0.007, *P *< 0.001, and *P *= 0.029, respectively; one-sided Binomial test). However, after Bonferroni correction, only the association for the COG "Amino acid transport and metabolism" was significant (Bonferroni corrected *P *= 0.004).

### Evidence for 57 *A. pleuropneumoniae *core genes subject to positive selection

The analyses of positive selection implemented in PAML was carried out for 1,587 core genome genes of *A. pleuropneumoniae *(in our initial experiment we included all 1,960 orthologous genes). Based on the LRT statistic for comparing the null model M1a and the selection model M2a with the  distribution and corrections for multiple testing (FDR < 20%), a total of 57 genes were identified to be under strong positive selected pressure (Table 3; Additional file 3). Genes in the COG category "General function prediction only" were significantly enriched (*P *= 0.004; one-sided Binomial test). Except for four positively selected genes in the COG category "cell wall/membrane biogenesis", many genes with homologues in other COG categories or without homologues in the COG collection were also predicted to encode proteins localized on surface/membrane and simultaneously subject to positive selective pressure, e.g. *gntT*, *cysW*, *apaA*, *pcaK*, *aphA*, *pqiB *and *ytfN*.

**Table 3:** Genes that show evidence for positive Darwinian selection

Name (Systematic)	Cluster ID ^a^	COG ^b^	Putative function of coding products	2Δℓ^c^	Q-value	p ^d^	*ω*^e^	Positively selected sites ^f^
*napA*	APO_0068	C	Periplasmic nitrate reductase	10.03	0.189	0.000	1.02	
*glpA*	APO_0197	C	Anaerobic glycerol-3-phosphate dehydrogenase subunit A	12.26	0.096	0.009	11.45	
*fumC*	APO_0306	C	Fumarate hydratase	15.63	0.032	0.004	99.56	
*prlC*	APO_0109	E	Oligopeptidase A	14.72	0.042	0.000	607.84	
*argH*	APO_0322	E	Argininosuccinate lyase	13.10	0.071	0.014	10.79	423
*gntT*	APO_0415	E	Transporter, gluconate/H+ symporter (GntP) family	11.74	0.106	0.008	13.97	190, 193
*proA*	APO_0424	E	Gamma-glutamyl phosphate reductase	10.02	0.189	0.051	3.29	
*artP*	APO_0998	E	Arginine transport ATP-binding protein	14.75	0.042	0.004	521.75	225
*leuD*	APO_1132	E	3-isopropylmalate dehydratase small subunit	11.14	0.135	0.032	57.70	
*cpdB*	APO_0120	F	2',3'-cyclic-nucleotide 2'-phosphodiesterase/3'-nucleotidase	19.13	0.010	0.032	3.82	9, 99, 554
*purD*	APO_0394	F	Phosphoribosylamine--glycine ligase	13.44	0.062	0.061	34.74	
*glgP*	APO_00f74	G	Maltodextrin phosphorylase	14.13	0.054	0.019	9.66	13, 27, 392
*kdgK*	APO_0752	G	2-dehydro-3-deoxygluconokinase	9.98	0.190	0.042	8.28	
*hemN*	APO_0325	H	Oxygen-independent coproporphyrinogen-III oxidase	21.87	0.005	0.015	27.37	38, 136, 428
*lipB*	APO_1067	H	Octanoyltransferase	10.14	0.189	0.000	1.00	
*valS*	APO_0044	J	Valyl-tRNA synthetase	22.18	0.005	0.016	3.82	47, 792
*rumA*	APO_0356	J	23S rRNA (uracil-5-)-methyltransferase	26.17	0.002	0.003	47.26	20
*trmA2*	APO_0540	J	tRNA (uracil-5-)-methyltransferase	10.70	0.160	0.028	4.08	24, 210
*yhgF*	APO_0081	K	Transcriptional accessory protein	18.60	0.010	0.024	3.78	10, 142
*lysR*	APO_0797	K	Transcriptional regulatory protein	18.79	0.010	0.012	10.60	284, 287, 292
*parC*	APO_0090	L	DNA topoisomerase 4 subunit A	13.47	0.062	0.003	10.50	357
*mutM*	APO_0873	L	Heptosyltransferase family	12.15	0.096	0.077	14.90	270
*tatD*	APO_0977	L	Deoxyribonuclease	13.73	0.059	0.198	2.42	188, 204, 234
*hcsA*	APO_0102	M	Capsule polysaccharide modification protein	10.07	0.189	0.027	3.16	483
*hcsB*	APO_0367	M	Capsule polysaccharide modification protein	18.72	0.010	0.034	18.52	13, 136, 366
*ompP2B*	APO_0561	M	Outer membrane protein P2-like protein	16.15	0.026	0.095	8.07	306, 317, 320
*mscL*	APO_1465	M	Large-conductance mechanosensitive channel	12.17	0.096	0.044	103.40	97
*ptrA*	APO_0039	O	Protease III	17.69	0.013	0.049	6.26	43, 44, 50, 91, 617, 909
*glnD*	APO_0064	O	uridylyltransferase	17.93	0.013	0.024	12.23	17, 190, 514, 551
*sppA*	APO_0149	O	Protease 4	10.42	0.173	0.030	9.37	
*lonH*	APO_0207	O	Lon protease	15.24	0.035	0.013	106.47	19
*nrfG*	APO_0983	O	Formate-dependent nitrite reductase complex subunit	12.29	0.096	0.084	27.77	
*tehA*	APO_0711	P	Tellurite resistance protein	11.70	0.106	0.007	70.81	252
*cysW*	APO_0883	P	Sulfate transport system permease protein	13.80	0.059	0.074	3.68	145
-	APO_0030	R	Helicase	12.14	0.096	0.051	2.56	
*pqiB*	APO_0055	R	Paraquat-inducible protein B	20.31	0.008	0.046	3.62	259, 318, 384
*tldD*	APO_0268	R	Protease	10.16	0.189	0.009	15.91	377, 379
-	APO_0269	R	Hypothetical protein	10.02	0.189	0.000	1.00	463
*pcaK*	APO_0359	R	Major facilitator transporter	12.09	0.096	0.022	5.96	195, 238, 416, 417
*thiH*	APO_0509	R	Thiazole biosynthesis protein	18.32	0.011	0.012	12.98	15, 55
*murQ*	APO_0754	R	N-acetylmuramic acid 6-phosphate etherase	17.69	0.013	0.059	4.05	54, 99, 293
-	APO_0792	R	Nucleoside-diphosphate sugar epimerase	11.92	0.102	0.060	9.71	
*rssA*	APO_0839	R	Patatin	11.22	0.132	0.019	41.55	225
*cof*	APO_0888	R	Hydrolases of the HAD superfamily	10.62	0.160	0.000	25.15	
*smtA*	APO_0969	R	Methyltransferase	10.66	0.160	0.045	101.87	134, 204
*aphA*	APO_1107	R	Membrane protein affecting hemolysin expression	10.83	0.153	0.012	24.96	46
*recX*	APO_1397	R	Regulatory protein	19.96	0.008	0.008	37.09	47
*ytfN*	APO_0025	S	Hypothetical protein	14.07	0.054	0.002	239.60	553
-	APO_0098	S	Oligopeptide transporter	20.79	0.007	0.012	15.91	82, 127, 546
*glnE*	APO_0041	T	Glutamate-ammonia-ligase adenylyltransferase	11.88	0.102	0.023	3.60	53
*typA*	APO_0150	T	GTP-binding protein	33.59	0.000	0.006	15.43	395, 567, 569
*apaA*	APO_0772	T	ABC transporter, periplasmic binding protein	13.49	0.062	0.031	7.10	103, 147
-	APO_0051	-	Hypothetical protein	19.00	0.010	0.088	5.16	105
*guaB*	APO_0259	-	Inosine-5'-monophosphate dehydrogenase	15.25	0.035	0.013	4.98	448
-	APO_0393	-	Hypothetical protein	24.92	0.002	0.027	57.62	97, 104, 134, 355
-	APO_0571	-	Hypothetical protein	12.27	0.096	0.020	28.21	199, 275, 283
*wecF*	APO_0577	-	TDP-Fuc4NAc:lipid II Fuc4NAc transferase	22.68	0.005	0.035	14.23	70, 92, 182, 183, 289

^a ^Protein designations were taken from the *A. pleuropneumoniae *ortholog annotation that was summarized in our recent publication [19].

^b ^The abbreviations of COG function categories were assigned based on Figure 1.

^c ^2Δℓ denote the statistic of likelihood ratio test.

^d ^The proportion of the amino acid sites under positive selection.

^e ^*ω *is equal to the ratio of *d*_N _to *d*_S _(Number of nonsynonymous changes per nonsynonymous sites/Number of synonymous changes per synonymous site) for amino acid sites under positive selection (model M2a).

^f ^Positively selected sites identified with posterior probability *P *> 95%.

Notably, there was no obvious discrepancy for the values of *d*_S _between genes under positive selection and the remaining genes; whereas the *d*_N _values together with the number of informative sites and genetic diversity were significantly higher in the positively selected genes (*P *< 0.001, *P *< 0.001, *P *= 0.023; one-sided U-test). No association between positive selection and COG categories was observed, as the number of positively selected genes is low in each role category.

Among 57 positively selected genes, 24 genes also showed significant evidence for homologous recombination detected by all four recombination tests. Furthermore, 41 genes under positive selection pressure showed evidence for recombination identified by at least one test. It indicates that positive selection should be associated with intragenic recombination, as recombination can lead to phylogenic incongruence and highly false positives when selective pressure on protein coding sequences was estimated [3,29].

## Discussion

Gene acquisitions and losses that contribute to the virulence and serotypic diversification of *A. pleuropneumoniae *have been depicted in detail [18,19], but our understanding on small genetic variations caused by positive selection and homologous recombination, which also factually influence the evolutionary trajectories of protein coding genes, has not been well considered for this swine pathogen so far. In this report, we chose 12 genomes of *A. pleuropneumoniae *to study the evolutionary driving forces acting on the core genome of this animal pathogen using a comparative phylogenomic approach.

### Intragenic recombination and positive selection both play a key role in the evolution of *A. pleuropneumoniae *pan-genome

Tests for intragenic homologous recombination and positive selection were performed with 1,587 orthologous genes present in the core genomes of twelve strains of *A. pleuropneumoniae*. Overall, our results indicated that about a quarter of the genes in *A. pleuropneumoniae *core genome showed significant evidence for intragenic recombination. In comparison, core-genome recombination was also evident in both species of the genus *Streptococcus*, as 18% and 37% of the core genome for *S. agalactiae *and *S. pyogenes*, respectively, showed evidence for homologous recombination [5]. Notably, in *A. pleuropneumoniae*, two COG categories "Replication, recombination and repair" and "Amino acid transport and metabolism", which both presented high values of *d*_S _and *d*_N_, were favored by intragenic recombination.

On the other hand, 57 *A. pleuropneumoniae *genes, accounting for approximately 3.6% of the core genome, were identified to be undergoing positive selection. Another similar study on the identification of genes under positive selection in *E. coli *reported that 0.7% of 3,505 genes found in six *E. coli *genomes showed evidence for positive selection and no evidence for recombination [1]. Like other pathogenic bacteria, a substantial number of positively selected genes in *A. pleuropneumoniae *encode protein products involved in the biogenesis and structural components of bacterial cell wall and/or outer membrane. These genes are likely to be associated with co-evolutionary arms races between pathogenic microorganisms and hosts. To further decipher the roles of evolutionary pressure operating on the core genome of *A. pleuropneumoniae*, we analyzed the functional properties of the positively selected genes and potentially important residues subject to positive selection.

### Genes subject to positive selection in *A. pleuropneumoniae*

We found that many protein products encoded by the positively selected genes were exposed on the cell surface or involved in structural constituents of bacterial cell wall. Some of these proteins have been reported to be important virulence factors associated with bacterial adherence, colonization and persistence. Therefore, it suggests that the genes under diversifying selection may dynamically interact with the host immune and defense systems.

**Figure 2:**
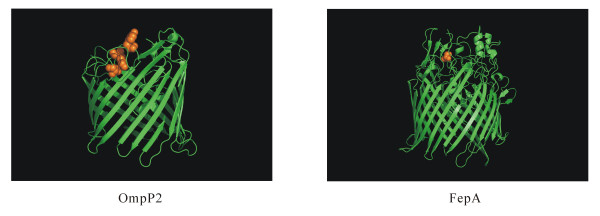
**Three-dimensional structural models of beta barrel porins OmpC and FepA**. Orange spheres stand for amino acid sites that are subject to strong positive selection (posterior probability > 95%).

The beta barrel porins are pore proteins that allow the passive diffusion of small, hydrophilic, or changed molecules across Gram-negative bacterial outer membranes [48]. The pore proteins have been believed to be crucial for not only dynamic interactions with the host immune system, but bacterial pathogenesis as well [1,49]. An outer membrane protein OmpP2, which was predicted to be beta barrel porin, showed strong evidence for positive selection with a low *q*-value (Table 3). The results of the Bayes empirical Bayes (BEB) analyses showed that *A. pleuropneumoniae *OmpP2 amino acid residues 306, 317, and 320 were subject to intense positive selective pressure (Figure 2). The three residues all located on a predicted extracellular loop in the C-terminus, perhaps associated with potential antigenic epitope. In addition, OmpP2 has been experimentally confirmed to be essential for *in vivo *survival of *A. pleuropneumoniae *by signature-tagged mutagenesis and also an immunogenic surface antigen by the immunoproteomic approach [50,51]. In our initial selection analyses using a set of 1,960 genes, gene *fepA *present in 11 *A. pleuropneumoniae *genomes encodes a beta barrel porin (Figure 2) and was also identified with evidence for positive selection (data not shown). FepA of *A. pleuropneumoniae *shared a common TonB-dependent receptor plug domain (PF07715) with *E. coli *outer membrane protein FepA that is a receptor for ferric enterobactin and for colicins B and D [52]. FepA of *A. pleuropneumoniae *has already been reported to exhibit immunogenic activity [53]. The adaptive changes in both porins might be beneficial for *A. pleuropneumoniae *to escape from the host immune systems and attack of phages, antibiotics, and colicins.

Bacterial surface polysaccharides, which are often involved in adherence and colonization, may be directly exposed to the host immune pressure. Three *A. pleuropneumoniae *genes (*hcsA*, *hcsB*, and *wecF*) participated in biogenesis of surface polysaccharides showed significant evidence for positive selection. The products of selected genes *hcsA *and *hcsB *code for capsule polysaccharide modification proteins that share 63% and 64% identity with *Haemophilus influenzae *HcsA and HcsB, respectively, which facilitate transport of capsular polysaccharide across outer membrane and are essential for bacterial virulence [54]. Besides, the positively selected gene *wecF *codes for a 4-alpha-L-fucosyltransferase and is located at a *wec *locus which has highly conserved colinearity in all *A. pleuropneumoniae *genomes. The products of *wecF *together with other *wec *genes exhibit high similarity to the *E. coli *K12 homologues that are involved in the assembly of a cell surface glycolipid [55].

The other gene *apaA *encoding an antigenic membrane lipoprotein that could provide cross-protection against heterologous *A. pleuropneumoniae *serovars [56], was also under strong positive selection (*q-value *= 0.062). The above analyses strongly demonstrated that the positively selected genes involved in the biosynthesis and structural composition of cell surface/wall have undergone adaptive functional changes, perhaps allowing bacterial pathogens to escape recognition by the host immune system and phages. Such phenomena have already been proposed by the previous studies of natural selection on the *E. coli *genome [1,14].

The proteases of *A. pleuropneumoniae *have been reviewed to be one of important virulence factors and contribute to pathogenesis [57]. Overall, 4 protease genes (i.e., *ptrA*, *lonH*, *sppA *and *tldD*) showed significant evidence for positive selection. The precise function of these protease genes identified here, to our knowledge, was not well understood for this pathogen. However, proteolytic enzymes are pivotal to the secretion processes of Gram-negative pathogens and several of them have been described as attractive drug targets in other pathogens, e.g. ClpP [58] and Lon [59].

## Conclusion

Our findings indicated that intragenic homologous recombination and positive Darwinian selection, unsurprisingly, indeed play crucial roles in the evolution of pathogenic *A. pleuropneumoniae*. In genes with extensive functional classification we found genes involved in the formation of cell surface/membrane are favored by the positive selective pressure. The adaptive changes in these positively selected genes and/or residues likely attribute to dynamic interaction caused by the host immune and defense systems. Of course, the diversifying selective forces of genes encoding metabolic functions may be also advantage for improving bacterial fitness in response to a variety of environmental signals. More experimental works are required for verifying the functions of these adaptive genes in future. Overall, the genetic evidence of positive selection will provide promising targets for further researches in the mechanisms of immune evasion and the host-pathogen interaction in *A. pleuropneumoniae*.

## Competing interests

The authors declare that they have no competing interests.

## Authors' contributions

ZX carried out the data collection, data analyses, wrote the manuscript. ZX and RZ participated in its design and revised the manuscript. RZ and HC supervised and coordinated the project. All authors read, edited and approved the final manuscript.

## Supplementary Material

Additional file 1 **Highly conserved genes in the core genome of *A. pleuropneumoniae***. Detailed information for individual gene alignment is provided, including nucleotide diversity, informative sites, and codon bias. Click here for file

Additional file 2 **Detailed information on test of recombination**. *A. pleuropneumoniae *genes showing evidence for recombination detected by at least one method (FDR < 10%). Click here for file

Additional file 3 **Alignments for positively selective genes**. Compressed file containing all alignments for genes under positive selection (FASTA format). Click here for file
